# How can occupational health and safety research adapt to the age of artificial intelligence?

**DOI:** 10.5271/sjweh.4340

**Published:** 2026-11-01

**Authors:** Arif Jetha, Julie Bowring

**Affiliations:** 1 Institute for Work & Health, Toronto, ON, Canada.; 2 Dalla Lana School of Public Health, University of Toronto, Toronto, ON, Canada.

Globally, employers across diverse industries are turning to artificial intelligence (AI) to innovate workplace processes and automate job tasks (1). Many of these AI applications are becoming increasingly independent from human operators and play prominent roles in making decisions and acting within the workplace. In this way, AI is contributing to shifts in work environments that may have significant implications for worker health, safety, and well-being (2). Governments are seeking to stimulate the commercialization of AI technologies and development of AI infrastructure to boost economic productivity (3). While there is an acknowledgment of the societal importance of creating safe, responsible, and pro-worker AI to mitigate risks that the technology may pose to workers and amplify its benefits, the health of workers has largely been absent from governmental agendas (4, 5). While there has been a growing focus by occupational health and safety (OHS) researchers on the impact of advanced digital technologies (6) and algorithmic management (7) on the health and safety risks faced by workers, studies of AI-specific transformations to the work environment and the health and safety of workers remain limited (8). Consequently, there is a paucity of evidence for the design of specific actionable strategies to protect and promote the health and safety of workers who are exposed to AI and AI-enabled systems in their working lives. Understanding how these technologies are transforming the relationship between work, health, and safety is an urgent scholarly priority for OHS researchers to address.

## Artificial intelligence at work

AI is an umbrella term for computing systems that perform tasks that have traditionally required human intelligence including recognizing patterns, making predictions, or generating decisions (9). Most modern AI systems rely on algorithms that learn patterns from large training datasets rather than following explicit, hand-coded rules. Machine learning, a core subfield, encompasses methods for this data-driven learning, with deep learning, a further subfield of machine learning that uses multi-layered neural networks, handling more complex patterns. Other subfields apply these techniques to specific data types, such as natural language processing (text) and computer vision (images and video).

Examples of AI adoption to perform specific job tasks exist in virtually all industries including those which have traditionally been open to adopting new technologies (eg, healthcare and manufacturing) and those less prone to technological change (eg, arts and culture) (10, 11). While in the early stages, AI is also being adapted to OHS activities within workplaces, such as in tools that monitor safety compliance in high-risk settings or promote healthy behaviors among workers (8). These examples have yet to show a mass disruption to the availability of jobs but can result in a re-organization of work between humans and intelligent machines that is fundamentally changing the way workers perform their jobs as well as their employment and work conditions (12). While research is scant, in table 1, we have synthesized available literature to describe how AI systems may impact different dimensions of the work environment such as work arrangements, and physical and psychosocial conditions.

**Table 1:** Artificial intelligence (AI) and its potential implications for the work environment.

Work environment dimension	Potential AI impacts	Workplace example
Employment status	AI-enabled automation of routine cognitive and manual tasks may displace workers into unemployment or force transitions into lower-quality jobs, with disparate effects across skill and demographic groups.	AI-powered robotic process automation could replace certain manufacturing positions.
Work arrangements	AI-driven job substitution could reduce the demand for full-time workers as employers restructure job roles around AI capabilities. At the same time, AI-related productivity gains could increase the demand for workers in certain fields and provide access to higher paying jobs.	Generative AI copilots that automate customer service tasks could prompt sales and services employers to redesign or reduce employees allocated to customer service roles.
Management of work	AI-based algorithmic management that directs, monitors, evaluates, and can deactivate workers in real-time, would alter the traditional employer–worker power relationship and could reduce worker autonomy. AI-related management could also contribute to data-driven decisions which boost job performance.	Ride-hailing and delivery platforms already use algorithms to assign tasks, set dynamic pay rates, and terminate accounts based on automated performance scores.
Working time	AI-driven scheduling and workflow-optimization tools may compress, intensify, or destabilize working hours and increase unpredictability for workers, even while improving employer efficiency.	AI-based dynamic staff-scheduling software is used in retail and logistics to match staffing levels to predicted demand in real time.
Physical	AI-guided collaborative robots (cobots) can reduce some manual strain and hazards while introducing new ones, such as human-machine collision risk, noise, and vibration from AI-controlled machinery.	AI-optimized order-picking and pacing systems can work alongside humans in warehouse fulfilment centres.
Chemical and biological	AI systems can be used as a hazard-control tool where sensors and predictive analytics can detect, forecast, and help mitigate chemical and biological hazards in the workplace earlier than traditional periodic monitoring.	AI-enabled biosurveillance systems can be used to flag infectious-disease outbreak risk in high-density workplaces such as meatpacking plants and healthcare facilities.
Psychosocial	Continuous AI-based monitoring, performance scoring, and surveillance can heighten job strain and reduce perceived autonomy, while AI decision-support tools may also reduce cognitive burden for some workers.	AI productivity-monitoring software (keystroke and webcam analytics, sentiment analysis) may be deployed in remote and call-centre work.

## OHS AI research challenges through the lens of the Partnership on AI and Quality of Work (PAIQ)

PAIQ is a seven-year project based at the Institute for Work & Health in Toronto, Canada that brings academic researchers together with multi-sectoral stakeholders to unpack how AI adoption impacts work and employment conditions and worker health and well-being (13). Drawing from our experience in PAIQ, we present three principal challenges that OHS researchers may face when engaging in research on AI in the workplace.

***Challenge 1: Rapid speed of AI advancement***. The applications and abilities of AI have and continue to change very rapidly with many frontier models surpassing human-level performance (14). Attributed to scalable architecture, improved computing power and infrastructure, and significant financial investment, AI has increased in its processing speed, ability to learn from diverse datasets, and capacity to perform tasks with increasing autonomy. AI advancements will contribute to an emerging number of applications within the workplace and thus ongoing changes to different dimensions of the work environment.

Many occupational hazards (eg, chemical, ergonomic, physical, and psychosocial) can have a fixed impact on work environments. In contrast, AI’s ability to learn and adapt may result in the technology constantly shifting its impact on the health and safety profile of an organization. These shifts may be exacerbated by AI advancement that can result in the technology reasoning and acting with a decreasing amount of human input. Keeping up with the pace of AI technological change is challenging, especially within the constraints of traditional time-intensive academic research protocols. There is a risk that the production of rigorous evidence will be obsolete by the time the findings are published.

To match the speed of AI advancement with the ability of OHS scholars to study its impacts, dynamic data collection approaches are needed, such as yearly scans of evidence sources to identify emerging threats and opportunities of AI in the workplace. As an extension of this strategy, PAIQ’s use of a living systematic review methodology allows for the tracking of peer-reviewed research on new forms of AI and their impact on work environments and different worker health outcomes (15).

***Challenge 2: AI invisibility and difficulties with primary data collection***. AI is commonly embedded in existing digital technology within a workplace such as automated vehicles, mobile phones, robots, or the Internet-of-Things devices, and its impact on working experiences is not always visible to workers encountering the technology (2). In the same vein, employers choosing to adopt AI systems may have less clarity on how the technology is functioning and affecting workers. Despite its potential invisibility, AI may be actively dictating work task performance and/or pace of work, monitoring workers’ performance, and making decisions. Workers and employers participating in OHS research may struggle to identify how AI is affecting different dimensions of work and employment conditions, creating challenges for primary data collection (eg, surveys or qualitative interviews). Therefore, OHS research can benefit from diverse disciplinary lenses—that is, from both computer and data sciences as well as the social sciences and health sciences—to better understand how AI design elements as well as workplace characteristics (eg, social structures, institutions, and power relations) intersect to contribute to AI’s impact on work environments and worker health.

***Challenge 3: AI upskilling for OHS researchers and practitioners***. Computer and data science are not typically part of the OHS researcher’s sphere of knowledge. OHS scholars may be unprepared to examine how algorithm design, data selection and training, and deployment decisions may play a specific role in changing work conditions and worker health. Lacking deeper AI knowledge creates challenges to proposing and pursuing research that can lead to the identification of AI hazards and understanding the mechanisms of harm. We also run the risk of developing applied solutions that do not address the salient challenges AI may pose to worker health within workplaces.

OHS researchers who gain greater familiarity with how different AI subtypes and systems behave, fail, and interact with humans on the job may be better equipped to study how the design, training, and application of AI can change different dimensions of the work environment and identify why and how the technology may positively or negatively impact worker health. The need for gaining expertise on AI may be particularly pronounced with the growing deployment of frontier systems that have the potential to more drastically change job performance and dimensions of the work environment.

## Concluding remarks

The adoption of AI at work creates new challenges and opportunities for the health, safety, and well-being of workers (16). The OHS sector offers a valuable perspective to the emerging discourse on AI safety and pro-worker AI by drawing greater attention to the intersection between the work environment and worker health (17). Importantly, OHS expertise has the potential to offer practical solutions that can be used to safeguard workers from novel AI-related hazards that may contribute to injury or illness. For OHS researchers to fully engage in the research and provide expertise to the AI research landscape, there is a need to transform the ways in which research is conducted to deploy dynamic research approaches, account for the invisibility of AI, and build AI literacy. While taking these steps requires an investment, they will enable OHS researchers to play a more prominent role in making an AI future healthier and safer for workers.
